# Prevalence and Risk Factors of Intestinal Parasite Infections in Greek Swine Farrow-To-Finish Farms

**DOI:** 10.3390/pathogens9070556

**Published:** 2020-07-10

**Authors:** Isaia Symeonidou, Panagiotis Tassis, Athanasios Ι. Gelasakis, Eleni D. Tzika, Elias Papadopoulos

**Affiliations:** 1 Laboratory of Parasitology and Parasitic Diseases, School of Veterinary Medicine, Faculty of Health Sciences, Aristotle University of Thessaloniki, 54124 Thessaloniki, Greece; isaia@vet.auth.gr; 2 Farm Animals Clinic, School of Veterinary Medicine, Faculty of Health Sciences, Aristotle University of Thessaloniki, 54627 Thessaloniki, Greece; ptassis@vet.auth.gr (P.T.); eltzika@vet.auth.gr (E.D.T.); 3 Laboratory of Anatomy and Physiology of Farm Animals, Department of Animal Science, School of Animal Biosciences, Agricultural University of Athens, 11855 Athens, Greece; gelasakis@aua.gr

**Keywords:** intestinal parasites, pigs, risk factors, Greece, swine farms

## Abstract

Intestinal parasites, helminths, and protozoa challenge health and welfare of pigs and deteriorate the sustainability of swine farms leading to monetary losses. A multicentric survey was conducted for approximately one year. Overall, 1150 fecal samples were collected from eight intensive farms in Greece at regular intervals and examined by flotation and Ziehl-Neelsen techniques. Age, season, and time of last recorded antiparasitic treatment were assessed as possible risk factors using binary regression models. The overall prevalence of intestinal parasitism in pigs was 44.7%. The most frequently detected parasites in the studied population were the protozoa *Balantidium coli* (37.8%), followed by *Entamoeba* spp. (8.3%), *Cystoisospora* *suis* (6.0%), and the nematodes *Ascaris suum* (3.7%), *Trichuris suis* (2.5%), and *Oesophagostomum* spp. (1.4%). Distribution of intestinal parasites in different age groups was as expected. In autumn, the prevalence of *Balantidium coli* infection enhanced whereas the prevalence of *Entamoeba* spp. and *Cystoisospora* *suis* infections increased in spring. Time of last recorded antiparasitic treatment influenced *Balantidium coli* and *Trichuris suis* infection levels. Our results demonstrated that swine intestinal parasitism in intensive farms of Greece seems to be relatively common and highlighted the importance of proper laboratory examinations, as well as the need for tailored made control programs.

## 1. Introduction

The vast majority of swine farms in Greece are farrow-to-finish intensive conventional units, with their own feed mill. Pigs remain permanently housed and under controlled environmental conditions, usually on slatted or semi-slatted floor, with limited access to outdoor areas, and thus rare contact with soil. However, partial access of breeding stocks to outdoor areas at some points of production (e.g., gestation units) is the norm, therefore, animals can be exposed to parasites.

Pigs may harbor numerous intestinal parasites, most commonly protozoa and nematodes. Although the course of such parasitic infections is usually subclinical, sometimes, clinical infections may occur particularly in growing pigs [1,2]. Various intestinal parasites have been implicated as causative agents of intestinal disorders such as vomiting, diarrhea, enteritis, typhlocolitis, and rectal prolapse, as well as general symptoms (e.g., anemia) and lesions (e.g., ‘white/milk spots’ on the liver) [3,4]. Additionally, parasitized pigs tend to be more susceptible to infectious and non-infectious diseases, which undermine their health and welfare status [3,5].

In many cases, the actual prevalence, transmission dynamics, and effects of intestinal parasites in pigs’ health, welfare, and production potential are underestimated. Moreover, there are well-known flaws and weaknesses of the commonly used preventive veterinary medicine programs in intensively reared pigs resulting from the scarcity of evidence-based antiparasitic protocols [6]. In modern conventional farms, parasitic diseases are not commonly diagnosed, and differential diagnosis of intestinal disorders rarely includes parasites as causative or contributing agents. This misperception is attributed to the notion that there are universally accepted antiparasitic programs, which sufficiently protect the animals, and the fact that parasitic infections are diagnosed more frequently in cases of extensively reared or organic swine farms [1,7].

It has been well documented that swine parasitism results in major monetary losses worldwide mainly due to reduced nutritional conversion and growth rate [1,2,5,8] and changes in body composition, i.e., less meat and heavier plucks [8,9]. It is noteworthy that some porcine parasites may pose a risk of infection to professionals involved in the value chain (e.g., farmers, veterinarians, and abattoir workers), either via direct contact or via exposure to contaminated environment [10,11]. The protozoon *Balantidium coli* can be transmitted from pigs to humans and act as an occasional pathogen [12,13] while *Entamoeba* spp. of swine origin does not seem to have a zoonotic implication [14]. *Cryptosporidium* is another zoonotic protozoon of pigs, which can cause serious diarrhea, particularly in immunodeficient individuals and children [11,15], whereas, *Ascaris suum* and possibly *Trichuris suis* may have public health implications [10,16]. 

To the best of our knowledge, there is a remarkable scarcity of large-scale, comprehensive studies on the occurrence and possible risk factors of pig intestinal parasitism in conventional swine farms in Greece. Therefore, the objectives of this repeated cross-sectional study were (i) to investigate the current epizootiological status of the major swine parasites, as has been shaped by the regularly applied antiparasitic programs in intensive farms in Greece the last decades; and (ii) to assess possible risk factors, which predispose to the aforementioned intestinal parasitic infections in intensive swine units.

## 2. Results

### 2.1. Epizootiology of Intestinal Parasites in the Studied Population

**Figure 1:**
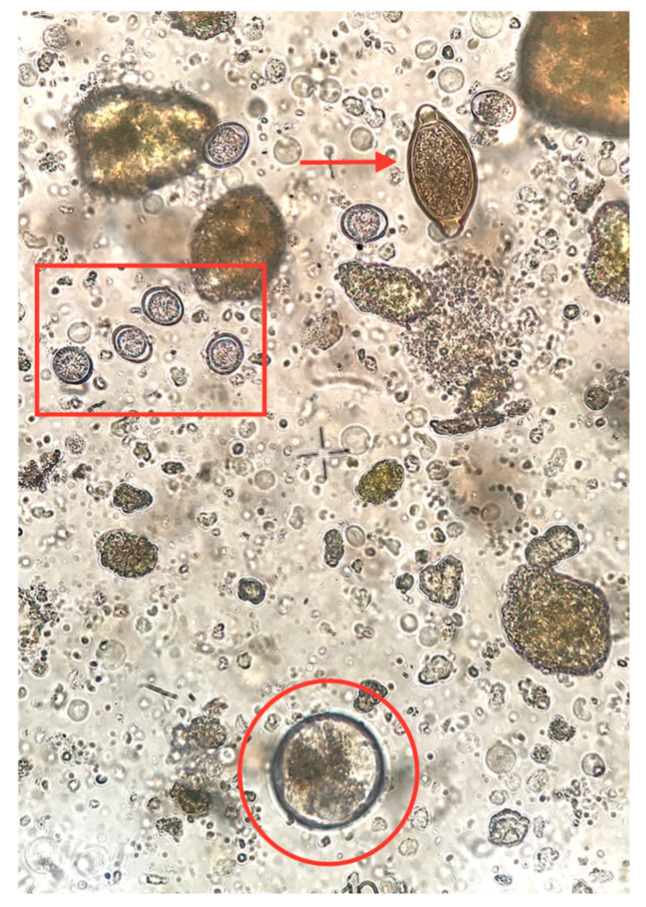
Various parasitic elements (×400 magnification) detected in pigs in Greece: *Trichuris suis* egg (arrow), *Cystoisospora suis* oocysts (rectangle), and *Balanditium coli* cyst (circle).

**Figure 2:**
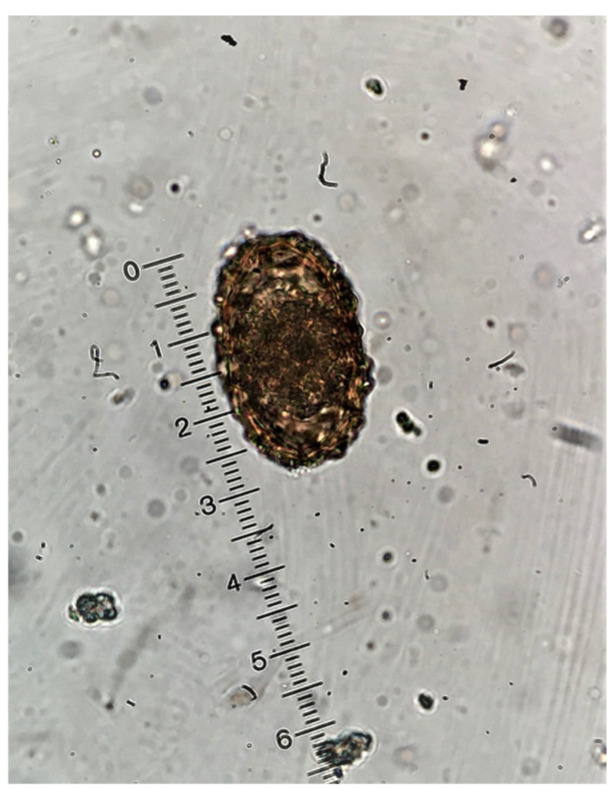
*Ascaris suum* egg as seen in the microscope (×400 magnification).

Diarrheic specimens were not observed in any of the age groups. The consistency of all stool specimens from all age groups, except from suckling piglets, was firm and the color was typical brown ranging from light brown to dark brown due to diet differences among groups. Fecal samples from suckling piglets were softer and of yellowish to light brown coloration due to diet (milk). In total, 44.7% (514/1150) of the examined samples were found to excrete at least one parasitic element. Among them, 374 pigs (32.5%) were found to be infected with only one parasite genera, while 112 (9.7%), 24 (2.1%), and 4 (0.3%) pigs were harboring two, three and four different parasite genera, respectively. Overall, infected animals were present in all eight farms, whereas six different intestinal parasite species were identified. *B. coli* was the most prevalent parasite (*n* = 435 pigs, 37.8%), followed by *Entamoeba* spp. (*n* = 95 pigs, 8.3%), *C. suis* (*n* = 69 pigs, 6.0%), *A. suum* (*n* = 42 pigs, 3.7%), *T. suis* (*n* = 29 pigs, 2.5%), and *Oesophagostomum* spp. (*n* = 16 pigs, 1.4%) (Figure 1 and Figure 2). The prevalence of the studied intestinal parasitic infections per group and per season is presented in Table 1 and Table 2, respectively.

### 2.2. Effects of Risk Factors on Parasitic Infections 

The effects of the studied risk factors used as independent variables into the regression models (season, age, time passed since the last antiparasitic treatment, and farm) for the six parasite genera (*Balantidium, Entamoeba, Cystoisospora, Ascaris*, *Trichuris*, and *Oesophagostomum*) are summarized below (Table 3).

#### 2.2.1. *B. coli*

*B. coli* parasitism increased as age progressed. In detail, suckling piglets and weaners had a 200 (*p* ≤ 0.001, CI 95%, 83.3 to 500.0) and a 33.3 (*p* ≤ 0.001, CI 95%, 20.0 to 55.6) times, respectively, lower probability of *B. coli* infection in comparison to sows. Similarly, growers and fatteners were 8.3 (*p* ≤ 0.001, CI 95%, 5.3 to 13.0) and 4.0 (*p* ≤ 0.001, CI 95%, 2.6 to 6.2) times, respectively, less likely to be infected by *B. coli* when compared to sows. Moreover, a higher likelihood of infection with *B. coli* was recorded in autumn (1.5 times, *p* ≤ 0.01, CI 95%, 1.1 to 2.2) than in spring. An anthelmintic treatment interval of less than 120 days was associated with a lower probability of *B. coli* infection (1.9 times, *p* = 0.004, CI 95%, 1.2 to 2.9 times). The effects of the variables used in the *B. coli* model are summarized in Table 3. The goodness-of-fit statistics in the *B. coli* model indicated good fit [χ^2^ (8 d.f.) = 7.202, *p* = 0.515]. A significant predictive value for *B. coli* infection was indicated by the omnibus test of model coefficients [χ^2^ (14 d.f.) = 487.938, *p* < 0.001]. Cox and Snell *R*^2^, and Nagelkerke *R*^2^ values were 0.346 and 0.471, respectively. 

#### 2.2.2. *Entamoeba* spp. 

Suckling and weaned piglets were not infected by *Entamoeba* spp. in any case. A lower probability of infection with *Entamoeba* spp. was recorded in autumn (2.0 times, *p* = 0.006, CI 95%, 1.2 to 3.4) and winter (2.4 times, *p* = 0.017, CI 95%, 1.2 to 4.9) than in spring. Table 3 shows the variables used for *Entamoeba* spp. model and their effects on *Entamoeba* spp. infection status. According to H-L test, the *Entamoeba* spp. model fitted well the data [χ^2^ (8 d.f.) = 6.922, *p* = 0.545]. Moreover, according to the omnibus test of coefficients the model was predictive of *Entamoeba* spp. infection [χ^2^ (10 d.f.) = 47.350, *p* ≤ 0.001]. Cox and Snell *R*^2^, and Nagelkerke *R*^2^ values were 0.040 and 0.093, respectively.

#### 2.2.3. *C. suis*

Growers and fatteners were not found to be infected by *C. suis* in any case, whereas only four cases oocysts of *C. suis* were detected in sows. The likelihood of *C. suis* infection tended to be lower (*ca*. 1.9 times) in winter than in spring (*p* = 0.083, CI 95%, 0.92 to 4.0); however, a significant difference was not concluded. The model provided a good fit to the data with the H-L test being insignificant [χ^2^ (8 d.f.) = 6.054, *p* = 0.641]. Moreover, according to the omnibus test of coefficients, the model was predictive of *C. suis* infection [χ^2^ (10 d.f.) = 28.279, *p* ≤ 0.01]. Cox and Snell *R*^2^, and Nagelkerke *R*^2^ values were 0.024 and 0.067, respectively.

#### 2.2.4. *A. suum*

Suckling and weaned piglets were not infected by *A. suum* in any case, and for that reason age was not used as a risk factor in the model. The effects of season and time passed since the last antiparasitic treatment were not significant. H-L test was statistically insignificant indicating that the model fits the data well [χ^2^ (8 d.f.) = 1.492, *p* = 0.993]. Furthermore, according to the omnibus test of coefficients the model had a significant predictive value of *A. suum* infection [χ^2^ (10 d.f.) = 48.792, *p* ≤ 0.001]. Cox and Snell *R*^2^, and Nagelkerke *R*^2^ values were 0.042 and 0.154, respectively.

#### 2.2.5. *T. suis*

Suckling piglets, weaners, and fatteners were not infected by *T. suis* in any case, whereas, in only one case *T. suis* eggs were identified in growers. The possibility of *T. suis* infection was 4.7 times lower for pigs with treatment intervals <120 days in comparison to those with treatment intervals >120 days (*p* = 0.032, CI 95%, 1.1 to 19.6). Table 3 summarizes the effect size of the variables used into the *T. suis* model. The H-L goodness-of-fit test in the *T. suis* model indicated good fit [χ^2^ (8 d.f.) = 1.843, *p* = 0.985]. A significant predictive value for *T. suis* infection was indicated by the omnibus test of model coefficients [χ^2^ (10 d.f.) = 43.380, *p* < 0.001]. Cox and Snell *R*^2^, and Nagelkerke *R*^2^ values were 0.037 and 0.176, respectively.

#### 2.2.6. *Oesophagostomum* spp.

Suckling piglets and weaners were not infected by *Oesophagostomum* spp. in any case, whereas only in two cases *Oesophagostomum* spp. eggs were found in both growers and fatteners. The model for the assessment of *Oesophagostomum* spp. infection did not fit the data well and therefore, its results cannot be considered reliable.

## 3. Discussion

The aim of this extensive study was to evaluate the prevalence and intensity of pig parasitesunder the pressure of regular antiparasitic treatments in intensive conventional units in Greece. Additionally, a second objective was to assess the risk factors involved and display their potential interactions in view of creating a clearer picture of swine intestinal parasitism in Greece. 

In total, the prevalence of intestinal infection with at least one parasite was ca. 45% (514/1150 pigs). Similar prevalence estimates have been recorded in previous studies conducted in other European countries. These studies have demonstrated that intestinal protozoa and helminths are commonly detected in swine farms in Germany [17,18], the Netherlands [19], the Nordic countries [5], Poland [20], and Switzerland [11] with reported prevalence, though ranging significantly among different countries. Evidently, in the present study, single infections were more frequent (32.5%, *n* = 374 pigs) than multiple infections (12.1%, *n* = 140 pigs). Moreover, protozoan infections were more prevalent (51.8%, *n* = 596 pigs) compared to helminthic ones (7.5%, *n* = 87 pigs), which is in accordance with the findings of Barbosa et al. [21]. 

*B. coli* was identified in all age groups and was the most commonly observed parasite in the studied swine population with a mean prevalence of 37.8%. This prevalence rate is within the range of the expected average prevalence reported in other large-scale surveys, which was from 30.6% to 57.1% of the examined pigs [2,11,22]. Regarding its taxonomy, although it was nominated, based on morphological differences, that isolates from pigs (*Balantidium suis*) were different species [23], genetic analysis of isolates from gorillas, humans, and pigs elucidated that only one species infects warm-blooded animals [24]. Pigs, along with wild boars, remain the main reservoirs of this cosmopolitan protozoon, which attracts interest due to its zoonotic implication [25]. Transmission from pigs to humans has been documented [12,13] and people working in proximity to pigs have a higher risk of acquiring balantidiosis [26]. In humans, these ciliates are opportunistic parasites and in immunocompromised individuals the disease may be severe [25], while in pigs they normally are non-pathogenic [26]. In some cases, *B. coli* has been previously implicated as an underlying factor of swine colitis, although this role has not been fully clarified [4]. Regarding risk factors, *B. coli* parasitism increased as age progressed. Similarly, Hindsbo et al. [22] recorded that the prevalence estimates significantly increased from 57% in suckling piglets to 100% in pigs older than 1 month and Morris et al. [27] reported that adult swine were more likely to be infected by this protozoon when compared to suckling piglets, weaners, growers, and fatteners. Correspondingly, Damriyasa and Bauer [18] also reported an age-related increase of balantidiosis in pigs. Furthermore, a higher likelihood of infection with *B. coli* was recorded in autumn than in spring. This can be presumably attributed to the fact that *B. coli* cysts survive best in humid ambience protected by direct sunlight [28]. Notably, the administration of an anthelmintic treatment in time interval of less than 4 months was associated with a ca. 2 times lower probability of *B. coli* infection. It has been established that helminths mediate generalized immunosuppression that abates immunity against protozoa [29]. It is therefore safe to speculate that the timely elimination of helminths alters the balantidiosis dynamics pattern in favor of the host.

Other protozoan parasites found in our study were *Entamoeba* spp. (*n* = 95 pigs, 8.3%). A study in Germany has recorded a quite similar prevalence of 14.0% for *Entamoeba* spp. infections in farrow-to-finish swine farms [18]. *Entamoeba* spp. in pigs are mostly harmless and not of epidemiological relevance in Europe [14] since pigs are not considered a reservoir of *Entamoeba histolytica* for humans [4]. In this study, the frequency of amoebae infection was higher in sows than growers and fatteners, which agrees with the findings by Damriyasa and Bauer, who surveyed swine farms in Germany [18]. Furthermore, our results suggested that pigs reared in autumn and winter had a lower probability of infection with *Entamoeba* spp.

Coccidia affected 6.0% of the examined animals (*n* = 69 pigs). Although several species of the genera *Eimeria* and *Cystoisospora* can infect pigs, *C. suis* (syn. *Isospora suis*) is the predominant coccidium [30,31]. *C. suis* may cause transient diarrhea, which is often implicated by secondary pathogens such as bacteria and viruses [32], thus resulting in weight loss and managerial costs [33]. Additionally, this protozoon can lead to possible alterations of intestinal epithelium and gut microbiota and consequent diminished nutrient absorption [4,33,34]. This apicomplexan protozoon affects mainly suckling piglets, which are unable to mount an adequate primary immune response [31,35]. Therefore, the increased prevalence of *C. suis* infections in our study, reported in suckling piglets followed by weaners, was an expected finding. 

In detail, approximately 1 out of 5 (19.1%) suckling piglets and 1 out of 10 (9.1%) weaners were found to be infected; whereas, only in four cases was *C. suis* infection found in sows. Age-dependence of *C. suis* infections has been confirmed by other surveys in Europe, which have reported an increasing immunity by age against *C. suis* [5,11,20,30,36]. The recorded prevalence for *C. suis* in suckling piglets vary from <1% to >40% (e.g., 10.0% in Germany [18], 12.8% in Switzerland [11], 20.9% in Southern Germany [36], 42.9% in Poland [20] and for the Nordic countries: Denmark 19.5%; Finland 4.5%; Iceland 31.8%; Norway 0.3% and Sweden 20.1%) [5]. The discrepancy in infection intensity with some of aforementioned studies might be explained by the fact that toltrazuril, an effective coccidiostat that suppresses oocyst excretion and enhances piglet health in cases of experimental infections [37] and in the field [38], was administered per os at the first days of age in all farms that were included in the current study. Moreover, a factor with potential effect on *C. suis* infection could be the season, however, this effect was not concluded by the analysis of our dataset. A rise in temperature after the cold winter season in Greece and a relative high humidity are considered favorable climatic conditions for increasing oocyst sporulation [39] and this may account for the relatively higher prevalence of coccidiosis recorded in spring. In other animals, such as goats [40] and broiler chicks [41], similar patterns for coccidial infections being detected more frequently in spring than in winter have been established.

*Cryptosporidium* spp. oocysts were not detected in the present study. Accordingly, in a large-scale survey in Germany, where feces of pigs were examined for a period of 10 years, oocysts of this protozoon were not found [42]. It should be highlighted that a very low number of oocysts are excreted in pigs’ feces, which accounts for the decreased sensitivity of microscopy for identification of this protozoon [43].

Regarding helminths, the species *A. suum* and *T. suis* and *Oesophagostomum* were detected. These nematodes have global distribution and occur in all kind of production systems [16]. High prevalence and infection intensity of intestinal nematodes are recorded in traditional and organic swine farms as opposed to low levels observed in intensive swine production units [42,44,45,46]. 

It should be noted that the host-specific status of both *A. suum* and *T. suis* are being debated [10,16]. *A. suum* is in close relation to *Ascaris lumbricoides* which affects approximately 1.2 billion people around the world. In the same frame, *T. suis* has been linked to *Trichuris trichiura,* that affects 795 million humans [47]. Experiments have confirmed that both of these nematodes can cross-infect pigs and humans [48,49]. Moreover, studies using credible molecular tools revealed the presence of shared cytochrome c oxidase subunit 1 (cox1) haplotypes between *Ascaris* of human and porcine origin [50,51]. Correspondingly, concerning *T. suis*, sequence analysis of the Internal Transcribed Spacer 2 (ITS-2) region of sympatric worms [52] and of the ITS region of eggs from worms collected from non-human primates and pigs [53] demonstrated that although *Trichuris* of the two hosts represent two different species, *T. suis* infection may be a zoonosis. Whether these two species are swine-specific pathogens or potentially zoonotic ones remains to be established by sophisticated molecular tools [10].

*A. suum* is the most prevalent helminth in pigs and its prevalence varies depending on farm management practices and geographical regions [20,44,46]. The mean prevalence of *A. suum* recorded in the present study (3.7%) was low, although it should be stressed that the actual infection rate might be much higher. A crucial feature of porcine ascaridiosis, which should be taken into consideration, is that a large proportion of pigs with latent infection do not excrete eggs in their feces while their serological response results are positive, thus, the actual infection rate is often underestimated [54].

Pigs infected with *A. suum* have reduced villar-height to crypt-depth ratio in the intestinal mucosa [55] and consequently display reduced feed utilization and impaired lactase activity in their gut [56]. Moreover, the hepato-tracheal migration of the larvae causes injury and subsequent inflammatory response leading to the formation of the characteristic white spots in the liver of infected pigs [57] as well as to the induction of respiratory distress accompanied by short dry coughs or even severe dyspnea in some cases [58].

As it was expected, *A. suum* eggs were not detected in suckling piglets and weaners due to the long prepatent period of this nematode [59]. In this study, the prevalence rate was lower for growers and fatteners than sows (0.9% in growers, 0.9% in fatteners, and 16.5% in sows). A survey in Denmark reported a similar pattern for *A. suum* infection, where the highest prevalence of parasitism by *A. suum* in industrialized swine farms was recorded in sows. An explanation for this infection pattern, according to the authors, is that no transmission occurs in the pens of growers and fatteners although the pens are contaminated [60]. This has been attributed to the lack of high relative humidity in indoor housing units [60], which is a prerequisite for egg embryonation and survival [61]. It has been demonstrated that desiccation causes the eggs to collapse before becoming infective [60]. On the other hand, the contamination is high under favorable environmental conditions that facilitate egg survival (e.g., in the outdoor areas of the pens) [62]. In addition, *A. suum* eggs that survive remain viable and infective for up to at least nine years [63] and therefore infections build up in sows as age progresses.

*T. suis* infection likelihood is directly linked to the housing system and although this nematode is widespread amongst pigs, it is sporadically found under intensive swine farming systems [5,11,44]. This is in accordance with the results of the present study where the total prevalence of the pig whipworm in all age groups was 2.5%.

Clinically evident cases of trichurosis in pigs, characterized by mucous diarrhea, are scarce and the underdiagnosed subclinical infections cause reduced growth rate [64]. Of note, *T. suis* interferes with the structural architecture and function of the large intestine and alters the porcine colon microbiota, thus increasing susceptibility to secondary pathogens [65,66]. In detail, a study on germ-free pigs has demonstrated that infection with *T. suis* acts as an immune modulating factor and enhances the pathology of *Campylobacter jejuni* [67]. In the same context, Shin et al. [68] observed, in a pig experimental model, that *C. jejuni* was pathogenic only in the concurrent presence of *T. suis*. Moreover, the swine whipworm has been suggested to offer an entry point for agents of the swine dysentery complex [3]. Another experiment in pigs displayed that whipworm-induced immunosuppression of the intestinal mucosa to resident microbiota contributed to the establishment of necrotic proliferative colitis [69].

In the current study, all *T. suis* cases were reported in sows with the exception of one case which was observed in growers. This predominant occurrence of *T. suis* in adult pigs has been confirmed in other studies as well [5,20]. The prevalence of trichurosis is higher in adult pigs as a result of the long prepatent period of this nematode [59] and its resistant eggs, which maintain their infectivity and viability for up to 11 years [63,70]. Interestingly, the probability of *T. suis* infection was 4.7 times lower for pigs with treatment intervals <4 months. This result indicates that anthelmintic drugs in sows feed can induce lower whipworm load if administered regularly at <4-month intervals.

*Oesophagostomum* spp. larvae were identified only in one farm (*n* = 16 pigs, 1.4%). A low intensity of parasitism is expected for these nematodes since it is known that *Oesophagostomum* free-living larvae cannot survive during the warm and dry summer [71], which is the case in Greece. Furthermore, infections occurred more frequently in sows (5.2%) than in growers (0.9%) and fatteners (0.9%). This distribution type is characteristic for *Oesophagostomum* spp. and has been attributed to the weak immunogenicity of these parasites [44] that results in higher worm burdens in adult animals [5,20]. In our study, it is impossible to draw safe conclusions regarding risk factors and prevalence of *Oesophagostomum* spp. infections by age due to the low number of cases.

Results of the present study support the observation that the total elimination of intestinal parasitism in pigs is difficult to accomplish, even if regular and systematic antiparasitic prophylaxis is implemented. This can be attributed to many factors: (i) longevity and the subsequent probability of re-infection of breeding animals, e.g., lactating sows, that act as reservoirs of parasites for other age groups [2,21]; (ii) possible weaknesses of the commonly used treatments schemes in intensively raised pigs [6] that lie upon the application of in-feed preventive substance administration, independent of sows’ bodyweight differences among age groups, that could cause under-dosing; (iii) inefficiency of prophylactic schemes which could be attributed to long time intervals (e.g., exceeding 6 months) between administration time points, in sows feed, that could create an ‘unprotected time window’ in breeding stock’s life; and (iv) the re-occurrence of nematodes in intensive pig rearing units, which is associated with the increased viability of the highly resistant *A. suum* and *T. suis* eggs [63].

In addition to a preventive program, administered at time periods that do not exceed 4 months, it is advisable to take caution regarding a proper facilities sanitation plan in such confined units (regular feces removal where applicable, and proper cleaning and disinfection schemes), the reduction of crate-to-crate contamination carried on boots or clothing, the avoidance of pets and rodents, which act as mechanical transmitters, as all these are critical points of prevention of intestinal parasitic infection outbreaks in swine farms. Undertaking such actions will reduce the infectivity of the environment, thus the parasitic burden will be minimized [4,6].

## 4. Materials and Methods

### 4.1. Sampling Process

The samplings took place between August 2017 and June 2018. All animals were apparently healthy, i.e., absence of obvious clinical signs prior to sampling and originated from eight intensive farrow-to-finish farms of Northern Greece. Fresh fecal samples were collected with a gloved hand individually during defecation to avoid feces contamination. In total, three samplings were performed in each farm approximately every 3 months, during a study period of 10 months to encompass seasonal variation. The same number of specimens was collected in each sampling occasion from randomly selected animals of all the groups in each of the studied farms, as follows: 10 samples from sows (5 in gestation and 5 in lactation), and 10 samples per group from suckling piglets (up to 4 weeks of age), weaners (4–10 weeks of age), growers (10–16 weeks of age), and fatteners (16 to 22–24 weeks of age). Thus, 50 samples were collected during each sampling occasion, resulting in a total of 150 specimens from each farm for the total study period (3 samplings/farm) except from one farm where only two samplings were feasible. Consequently, a total of 1150 fecal samples were included in the study.

The capacity of conventional farms that were enrolled in the study, varied from approximately 250 to 1000 sows (from first to sixth parity). Breeding was performed with artificial insemination in the gestation unit and access to external areas was permitted after ultrasonographic confirmation of gestation on the fourth week after insemination. All participating farms applied preventive antiparasitic treatments on a regular basis in breeding stocks and suckling piglets. The norm was the administration of a macrocyclic lactone (i.e., ivermectin) in sows’ feed twice (7 farms) or three times (1 farm) per year, as well as in the feed of 10–12 weeks old growers (every 3 and 4 months in 4 farms and 4 farms, respectively). Additionally, in all farms, a coccidiostat (i.e., toltrazuril) was used per os at suckling piglets the first three days of age. Animals were randomly selected and information regarding season, age, and time period since the last antiparasitic treatment were registered for each sampling occasion. Specimens were placed individually in plastic containers, labeled, stored at 2–6 °C and transferred to the Laboratory of Parasitology and Parasitic Diseases of the School of Veterinary Medicine in Thessaloniki, where they were processed within 48 h.

### 4.2. Coprological Methods Used

Initially, each specimen was macroscopically examined to specify the macroscopic appearance of feces (consistency, color, etc.) and to detect the possible presence of adult nematodes. Thereafter, for each sample, the flotation technique was carried out as described by Faust et al. [72]. In brief, approximately 1 g of feces was diluted with tap water, passed into a centrifuge tube through a sieve (no. 150) and centrifuged at 200× g for 3 min. The supernatant was discarded and ZnSO_4_ solution (33.2% *w/v*, specific weight 1.3) was added to the sediment. The sediment was thoroughly diluted and ZnSO_4_ solution was added to just over the top of the tube so as to form a meniscus. A cover slip was placed on the top of the meniscus and following centrifugation of the tube at 150× g for 1 min, the cover slip was removed, placed on a microscope slide and examined under the optical microscope at ×100 and ×400 magnification.

For detection of *Cryptosporidium* spp. oοcysts, approximately 1 g of feces was diluted in tap water, passed through a sieve (no. 150) and centrifuged at 200× g for 3 min. The supernatant was discarded and drops of the aqueous sediment were coated on slides, the smears were stained with the Ziehl-Neelsen method [73] and examined under the optical microscope at ×1000 magnification.

Coproculture was conducted for obtaining infective larvae and identifying Strongylida eggs to the genus level. Parasitic elements were identified based on morphological characteristics [74,75]. A pig was considered infected if at least one parasitic element (oocyst, cyst, egg, larva) was observed.

### 4.3. Data Handling-Statistical Analyses

Six binary logistic regression models were used to test the possible relationship between sampling season (three levels; autumn, winter, spring), age (five levels; sows, suckling piglets, weaners, growers, fatteners), anthelmintic treatment interval (two levels; ≤4 months and >4 months), and farm (eight levels; farms 1 to 8) and the likelihood that a pig is infected with the most prevalent parasites, namely, *A. suum*, *T. suis*, *C. suis*, *B. coli*, *Entamoeba* spp., and *Oesophagostomum* spp.

Statistical significance of individual predictors was tested using the Wald χ^2^ statistic of their regression coefficients (βs). Goodness-of-fit for each individual model was assessed using the Hosmer-Lemeshow (H-L) test, as well as Cox and Snell R^2^ and Nagelkerke R^2^ indices.

### 4.4. Ethics Approval and Consent to Participate

The study was conducted in compliance with the national animal welfare regulations, i.e., the Presidential Decree 56/13 “Bringing Greek legislation into line with Directive 2010/63/EC of the European Parliament and of the Council of 22nd September 2010 (L 276/33/20.10.2010) regarding the protection of animals used for experimental and other scientific purposes”. The applied diagnostic veterinary procedures are not within the context of relevant EU legislation for animal experimentations (Directive 86/609/EC) and may be performed in order to diagnose animal diseases and improve animal welfare. No suffering was caused during sample collection. Consent was ensured by farm owners.

## 5. Conclusions

To the best of our knowledge, this is the first time the prevalence of intestinal parasitism of pigs reared in conventional intensive Greek swine farms is assessed. Our observations suggest that intestinal parasitism in Greek swine farms is present and should not be underestimated when planning preventive measures to mitigate the occurrence of pathogens at a farm level, even though the likelihood of severe clinical disease is moderate to low under current farm conditions. Moreover, intestinal parasites should be considered in the differential diagnosis of intestinal disorders as underlying factors or co-infection intestinal pathogens, even in swine farms with an ongoing preventive antiparasitic program. The presence of parasite genera with zoonotic potential indicates that professionals in the Greek pig industry may be at risk of exposure to them.

## Figures and Tables

**Table 1:** Prevalence of intestinal parasitic infections per group in the studied pig population (suckling piglets, weaners, growers, fatteners, sows, *n* = 230 pigs per group, total = 1150 pigs)

Parasite	Suckling Piglets	Weaners	Growers	Fatteners	Sows	Total
*Balantidium coli*	6 (2.6%)	31 (13.5%)	86 (37.4%)	125 (54.3%)	187 (81.3%)	435 (37.8%)
*Entamoeba* spp.	0 (0.0%)	0 (0.0%)	3 (1.3%)	8 (3.5%)	84 (36.5%)	95 (8.3%)
*Cystoisospora suis*	44 (19.1%)	21 (9.1%)	0 (0.0%)	0 (0.0%)	4 (1.7%)	69 (6.0%)
*Ascaris suum*	0 (0.0%)	0 (0.0%)	2 (0.9%)	2 (0.9%)	38 (16.5%)	42 (3.7%)
*Trichuris suis*	0 (0.0%)	0 (0.0%)	1 (0.5%)	0 (0.0%)	28 (12.2%)	29 (2.5%)
*Oesophagostomum* spp.	0 (0.0%)	0 (0.0%)	2 (0.9%)	2 (0.9%)	12 (5.2%)	16 (1.4%)
Single infection	44 (19.1%)	48 (20.9%)	83 (36.1%)	119 (51.7%)	80 (34.8%)	374 (32.5%)
Double infection	3 (1.3%)	2 (0.9%)	4 (1.7%)	9 (3.9%)	94 (40.9%)	112 (9.7%)
Triple infection	0 (0.0%)	0 (0.0%)	1 (0.4%)	0 (0.0%)	23 (10.0%)	24 (2.1%)
Quadriple infection	0 (0.0%)	0 (0.0%)	0 (0.0%)	0 (0.0%)	4 (1.7%)	4 (0.3%)

**Table 2:** Prevalence of intestinal parasitic infections in the studied pig population (*n* = 1150 pigs) per season

Parasite	Autumn (*n* = 400)	Winter (*n* = 350)	Spring (*n* = 400)
*Balantidium coli*	157 (39.3%)	141 (40.3%)	137 (34.3%)
*Entamoeba* spp.	25 (6.3%)	23 (6.6%)	47 (11.8%)
*Cystoisospora suis*	26 (6.5%)	13 (3.7%)	30 (7.5%)
*Ascaris suum*	13 (3.3%)	16 (4.6%)	13 (3.3%)
*Trichuris suis*	7 (1.8%)	9 (2.6%)	13 (3.3%)
*Oesophagostomum* spp.	0 (0.0%)	10 (2.9%)	6 (1.5%)

**Table 3:** Regression coefficients of the variables used in the models for the detected intestinal parasites

		B ^1^	S.E. ^2^	*P*	Odds Ratio	95% C.I.^3^ for EXP(B)	
						Lower	Upper
***Balantidium coli***	Autumn	0.43	0.19	0.023	1.53	1.06	2.21
	Winter	0.15	0.22	0.477	1.17	0.76	1.78
	Spring	*Ref.*					
	Suckling piglets	−5.30	0.454	0	0.01	0	0.01
	Weaners	−3.51	0.267	0	0.03	0.02	0.05
	Growers	−2.11	0.226	0	0.12	0.08	0.19
	Fatteners	−1.39	0.222	0	0.25	0.16	0.39
	Adult	*Ref.*					
	ATI (<120 days)	−0.65	0.223	0.004	0.53	0.34	0.81
	ATI (>120 days)	*Ref.*					
***Entamoeba* spp.**	Autumn	−0.72	0.262	0.006	0.49	0.29	0.82
	Winter	−0.87	0.364	0.017	0.42	0.21	0.86
	Spring	*Ref.*					
	ATI (<120 days)	−0.24	0.362	0.501	0.78	0.39	1.59
	ATI (>120 days)	*Ref.*					
***Cystoisospora suis***	Autumn	−0.10	0.293	0.733	0.91	0.51	1.61
	Winter	−0.65	0.373	0.083	0.52	0.25	1.09
	Spring	*Ref.*					
	ATI (<120 days)	−0.26	0.357	0.462	0.77	0.38	1.55
	ATI (>120 days)	*Ref.*					
***Ascaris suum***	Autumn	0.27	0.47	0.562	1.31	0.52	3.3
	Winter	−0.15	0.633	0.814	0.86	0.25	2.98
	Spring	*Ref.*					
	ATI (<120 days)	−0.97	0.753	0.2	0.38	0.09	1.67
	ATI (>120 days)	*Ref.*					
***Trichuris suis***	Autumn	0.06	0.606	0.921	1.06	0.32	3.49
	Winter	−0.85	0.59	0.151	0.43	0.14	1.36
	Spring	*Ref.*					
	ATI (<120 days)	−1.55	0.723	0.032	0.21	0.05	0.88
	ATI (>120 days)	*Ref.*					

^1^ B: Regression coefficient, ^2^ S.E.: Standard error, ^3^ C.I.: Confidence interval, ^4^ *Ref.*: Reference category, ATI: Anthelmintic treatment interval.
